# Neurological complications of monkeypox viral infection: a case of encephalitis

**DOI:** 10.1097/JS9.0000000000000138

**Published:** 2023-03-03

**Authors:** Olivier Uwishema, Christin Berjaoui, Ahmad Sabalbal, Tania Chaib, Khaled Abou Dib, Nouran Al Serw, Sarah El Kassem, Aghati El Ghazzawi, Muna Sleem, Abubakar Nazir

**Affiliations:** a Oli Health Magazine Organization, Research, and Education, Kigali, Rwanda; b Clinton Global Initiative University, New York, New York, USA; c Faculty of Medicine, Karadeniz Technical University, Trabzon, Turkey; d Faculty of Medicine and Surgery, Beirut Arab University, Beirut, Lebanon; e Department of Medicine, King Edward Medical University, Lahore, Pakistan

Highlights

- The presence of lymphadenopathy distinguishes monkeypox from smallpox, and the subsequent complications include encephalitis, bronchopneumonia, secondary infections, sepsis, corneal infections, and vision loss.
- Viral encephalitis, seen in various countries in association with monkeypox, can cause various neurologic complications ranging from altered mental status to seizures and focal neurologic deficits.
- There should be campaigns to raise knowledge of the clinical symptoms, transmission pathways, diagnosis, consequences, and prevention of this virus. Further prevention efforts should be made by the ministry of health, especially in endemic regions.

Monkeypox virus (MPX), which is one of the four pathogenic species affecting humans along with cowpox, vaccinia, and variola viruses, belonging to the *Orthopoxvirus* genus in the family Poxviridae, is considered to be the causative agent of the advancing zoonotic disease MPX^1^. Once the virus enters the body from any route, oropharynx, nasopharynx, or intradermal, it will replicate at that respective site and then spread to local lymph nodes. Clinically, MPX has an incubation period of 1–2 weeks^2^. The prodrome illness then begins with a fever of no more than 40.5°C. Patients usually complain of headache, malaise, and typical lymph node enlargement, particularly, the mandibular, cervical, or inguinal nodes^1^,^3^. The lesions persist between 2 and 4 weeks and develop at the same stage. They are centrifugal, umbilicated, and hard^2^,^3^. Despite the fact that the detailed dermatological manifestations are abundantly found in the literature, the neurological complications of this viral infection have yet to be properly documented and characterized. However, MPX has been reported to affect the central nervous system (CNS) with encephalitis being the most common neurological complication^3^,^4^. Furthermore, due to overlapping similarities between MPX and smallpox viruses, we can propose that the neurological complications of this viral infection can be significant. For instance, encephalopathy is the most common complication of smallpox infection, and while encephalitis, seizures, and strokes are less commonly seen, they are still documented following both viral smallpox-related infections as well as vaccinations with *Vaccinia* 5. Although rarely stated in the literature, encephalitis associated with MPX infection seems to behave similarly to viral encephalitis. Thus, MPX-associated encephalitis can infect the CNS retrogradely via nerve terminals or haematogenous dissemination. Once the virus enters the CNS, it replicates and disrupts neuron cell function, leading to cerebral edema, vascular congestion, and intracranial hemorrhage^6^. Clinically patients with encephalitis usually present with altered mental status, changes in the level of consciousness, personality changes, generalized or partial seizures, or focal neurological findings. Electroencephalographymight also show epileptiform changes^7^. Also, cerebrospinal fluid analysis of a patient with MPX-associated encephalitis may show MPX-specific immunoglobulin M antibodies^8^. It should be noted that patients with encephalitis might die or require admission to the ICU and even mechanical ventilation^4^. The best way to prevent viral encephalitis is to prevent viral infection. For instance, prevention from direct contact with primates and rodents, prevention from blood exposure and avoiding undercooked meat are ways to prevent transmission of the virus from its animal reservoir to humans^6^. In healthcare settings, infection-control measures should be implemented to prevent human-to-human transmission, such as proper sanitation and isolation of infected patients (Fig. 1). Vaccines against the smallpox virus, ACAM2000, have shown crossed immunity against the MPX virus^8^. Furthermore, the United States of America Food and Drug Administration has approved JYNNEOS, a vaccine against both smallpox and MPX viruses, for adults that are at high risk of infection. The Centers for Disease Control and Prevention recommends prophylaxis for those with a higher risk of exposure to the virus, however widespread vaccination during the 2022 outbreak is not recommended^9^. Lastly, the management of MPX depends on the severity. The majority of patients have mild disease. Mild cases require conservative management comprising fluid replenishment, antipyretics, and oxygenation^10^. However, patients who have or are at risk of dehydration, those who necessitate pain management, and those experiencing severe disease or complications, require hospitalization, and supportive care. For more severe complications, antiviral therapy may be indicated for patients with or at risk of severe disease (i.e. <8 years of age, breastfeeding and pregnant women, patients suffering from complicated infections, as well as immunocompromised patients), as well as patients with the infection affecting atypical sites (i.e. mouth, eyes, and genital area) 11.

**Figure 1:**
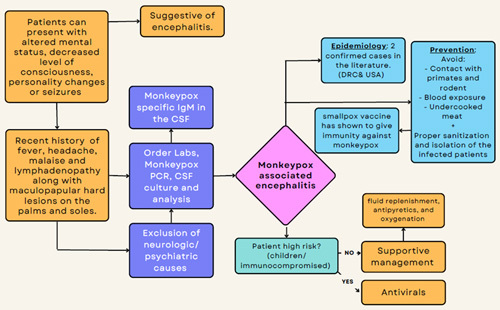
Summary – neurological complications of monkeypox viral infection, presentation, diagnosis, and management. CSF indicates cerebrospinal fluid; DRC, democratic republic of congo; IgM, immunoglobulin.

## Ethical approval

Not applicable.

## Sources of funding

None.

## Author contribution

O.U.: conceptualization, project administration, and writing – review and designing. Figure 1 was drawn and analyzed by author K.A.D. Manuscript writing, data collection and assembly, and final approval of manuscript by all authors

## Conflicts of interest disclosure

Not applicable.

## Guarantor

Abubakar Nazir, Oli Health Magazine Organization, Kigali, Rwanda. E-mail: abu07909@gmail.com. ORCID ID: 0000-0002-6650-6982
